# Associations between hydration state and pregnancy complications, maternal-infant outcomes: protocol of a prospective observational cohort study

**DOI:** 10.1186/s12884-020-2765-x

**Published:** 2020-02-07

**Authors:** Na Zhang, Fan Zhang, Su Chen, Feng Han, Guotian Lin, Yufei Zhai, Hairong He, Jianfen Zhang, Guansheng Ma

**Affiliations:** 1 Department of Nutrition and Food Hygiene, School of Public Health, Peking University, 38 Xue Yuan Road, Hai Dian District, Beijing, 100191 China; 2 Laboratory of Toxicological Research and Risk Assessment for Food Safety, Peking University, 38 Xue Yuan Road, Hai Dian District, Beijing, 100191 China; 3 Department of Nutrition and Food Hygiene, School of Public Health, Hainan Medical University, 3 Xue Yuan Road, Longhua District, Haikou, 100191 China; 4 Department of Obstetrics, The First Affiliated Hospital of Hainan Medical University, 31 Long Hua Road, Haikou, 100191 China; 5 Department of Laboratory, The First Affiliated Hospital of Hainan Medical University, 31 Long Hua Road, Haikou, 100191 China

**Keywords:** Hydration, Water intake, Pregnancy complications, Maternal and infant outcomes

## Abstract

**Background:**

Water requirements increases with gestational age. Insufficient water intake causes dehydration, which may adversely affect maternal health and birth outcomes. However, few related studies have been conducted. The purposes are to assess the water intake and hydration state among pregnant women, and to investigate the associations with pregnancy complications and maternal and infant outcomes.

**Methods:**

A prospective observational cohort study will be applied. A total of 380 pregnant women will be recruited from the First Affiliated Hospital of Hainan Medical University. Hydration biomarkers and health outcomes will be tested during 15~17 weeks’ gestation, 20~22 weeks’ gestation, 30~32 weeks’ gestation, during childbirth and 42 days after childbirth. Daily fluid intake will be collected using a *24-h fluid intake record for 7 consecutive days*. *A semi-quantified food frequency method* will be used to assess food intake and water intake from food. Anthropometric measurement will be taken following standardized processes. Intracellular fluid (ICF) and extracellular fluid (ECF) will be measured using a body composition analyzer. Morning fasting urine and blood osmolality will be tested by laboratory physicians using an osmotic pressure molar concentration meter. Pregnancy complications will be assessed and diagnosed throughout pregnancy and childbirth. Maternal-infant outcomes will be monitored using related indicators and technologies. In order to explore the internal mechanism and interactions from the perspective of endocrine, pregnancy related hormones (estradiol, prolactin, progesterone) and the hydration-related hormones (copeptin) will be tested during pregnancy. A mixed model of repeated measures ANOVA will be analyzed using SAS 9.2.

**Results:**

The results may provide basic data on water intake among pregnant women. The association between hydration state and maternal-infant outcomes will also be explored.

**Conclusions:**

This preliminary exploratory study findings will fill the gaps in the research on water intake, hydration and maternal health, birth outcomes, provide scientific reference data for updating recommendation on water adequate intake among pregnant women, and provide suggestion for developing water intake interventions.

**Trial registration:**

The protocol has been registered on the website of *Chinese Clinical Trial Registry*. The Identifier code is ChiCTR1800019284. The Registry date is 3 November, 2018. Registry name is “*Study for the correlation between hydration state and pregnancy complications, maternal and infant outcomes during pregnancy*”.

## Background

Water participates in a variety of physiological functions, and is essential for body development and survival [1–3]. Three sources of water input are available: drinking water, water from food and endogenous water. Four ways of water-output includes urine through urinary system, sweat through skin surface, breath through respiratory system, and feces through digestive system. Under normal conditions, water maintains a state of dynamic balance in body, that is, the amount of water-input is approximately equal to the amount of water-output [2, 3]. However, too much or insufficient water intake disturbs the dynamic water balance, changes the hydration state, and affects body health negatively. When water intake exceeds the regulatory capacity of kidney, it may cause acute water intoxication and hyponatremia. Insufficient water intake may induce a dehydrated state, which reduces cognition ability [4–6] and physical activity ability [7–10] and increases the risk of urinary system diseases (such as kidney stones, urinary tract infections and chronic kidney disease) [11, 12] and cardiovascular disease [13]. Therefore, maintaining an optimal hydration status is vital for human health.

During pregnancy, physiological changes cause daily water requirements to increase compared with people in normal physiological stages. Blood volume of pregnant women gradually increases from 6 to 8 weeks of pregnancy and reaches a peak at 32 to 34 weeks’ gestation. Water is the main component of human tissue, and 83% of blood is composed of water. Many changes occur in the urinary system: the kidneys become slightly larger; renal plasma flow and glomerular filtration rate increase in early pregnancy and remain at the high level throughout the whole pregnancy; and urine volume increases when being in the supine position and during the night [14]. Urination is the main mode of water output [15]. In the respiratory system, the ventilation increases by 40% /minute and the tidal volume increases by 39%, thus increasing the amount of water output through expiration. Under normal circumstances, approximately 500 mL of water per day is lost through sweating [15]. During pregnancy, water loss through sweating increases due to hyperactive adrenal and thyroid functions, an accelerated metabolism and increased cutaneous circulation. Nutrients and energy requirements for pregnant women are also increase, so food intake is increased. Water is the carrier of food metabolism, digestion, absorption, circulation and excretion. The water requirement is 1 mL for every 4.184 kJ of energy consumption, as much, more energy intake requires more water intake correspondingly [15, 16].

Surveys on fluid intake have shown that pregnant women have insufficient water intake. According to the data of the 1977–1978 Food Consumption Survey conducted by the US Department of Agriculture National, it was found that the average total water intake among 188 pregnant women was 2100 L/d [17]. In a study in New Zealand in 2014, the average daily water intake among 504 pregnant women was 2200 mL/d [18]. In a 2016 survey of 20 pregnant women, the average total water intake was almost 2259 mL/d [19]. In a 2016 study in Indonesia, the average daily water intake among 300 pregnant women was 2332 mL/d, although in approximately 42% of participants, the water intake was less than the recommended level (2048 mL/d) [20]. In China, only a few surveys on fluid intake among children and adults have been conducted. These studies have shown that only one third of the participants had adequate water intake and that almost one quarter of participants were dehydrated [21, 22]. However, no surveys has been conducted for water intake among pregnant women in China.

Changes occurring in the endocrine system during pregnancy may affect hydration. Prolactin begins to increase gradually at 7 weeks of pregnancy to promote mammary gland development for postpartum lactation. Aldosterone secreted by the outer globular band during pregnancy has a four-fold times. The secretion of estradiol at the end of pregnancy is 100 times greater than that in non-pregnant women [16]. Water balance is affected by various hormones. Insufficient water intake increases the secretion of vasopressin and aldosterone, which affects the water permeability of the distal renal tubules and collecting ducts. As a result, water reabsorption increases and water output decreases [23]. Copeptin, a stable peptide derived from the vasopressin precursor, is a potential predictor in various chronic diseases, such as diabetes insipidus and cardiovascular diseases [24]. Endocrine indexes related to hydration and pregnancy interact; thus, hydration state may be associated with pregnancy complications and maternal and infant outcomes.

Few studies have explored the associations of fluid intake and hydration state with pregnancy complications and maternal/infant outcomes. A prospective study in Italy involving 173 pregnant women revealed that total body water and intracellular and extracellular fluid in healthy pregnant women increased gradually throughout pregnancy, but an opposite trend was observed among women with gestational hypertension [25]. In a study conducted in Canada, the body compositions of 196 women 4 and 12 h postpartum were measured: the total body water of women without arterial hypertension was 44 L, whereas that of women with arterial hypertension was only 18 L [26]. This suggested that associations might exist between water retention and the development of hypertension. A US study showed that total body water may be a strong predictor of pre-eclampsia [27]. A Mexican study suggested that total body water in pregnant women with gestational hypertension and severed eclampsia was relatively higher than that in healthy pregnant women. In addition, chronic hypovolemia induced by insufficient water intake may be among the main risk factors in the development of diabetes [28], and higher intake of boiled water may reduce the risk of diabetes [29]. Data from the US Health and Nutrition Survey (2009–2012) showed that people without optimal hydration have a greater risk of obesity than those with optimal hydration [30], suggesting that hydration influences total gestational weight gain and postpartum weight retention. One study showed that the secretion of breast milk in lactating women increased with the amount of water intake [31]. A case–control study in the United States suggested that insufficient fluid intake was a risk factor for abortion requirements and preterm births, as well as low birth weight [32]. Other studies have shown that total body water has significant effects on birth weight [26, 33–36]. A US case-control study suggested that insufficient fluid intake was an independent risk factor of low birth weight [32]. In a randomized controlled trial in California, among 40 women with normal amniotic fluid index at 28 weeks of gestation, the amniotic fluid index increased by approximately 16% in the water intervention group and approximately 8% in the control group [37]. The relationship between the state of hydration during pregnancy and the physical infant outcomes has not been fully valued and studied, so there are not so many related literatures. More studies are needed to be carried out to explore the associations between water intake, hydration state and on pregnancy complications, maternal-infant outcomes.

In order to promote enough water intake, it is necessary to propose recommendations on water intake for pregnant women. Only some countries or organizations have made specific recommendations on water intake for pregnant women: 2.7 L/d is recommended by the American Institute of Medicine and 4.8 L/d is advised by the World Health Organization (WHO) [15]. A survey of dietary guidelines in 84 countries revealed that only 8 countries had quantitative recommendations for fluid intake [38]. Of the four editions of *Dietary Guidelines* (1989, 1997, 2007, and 2016) released in China, the first two editions had no recommendation for fluid intake [39, 40]. In 2007, the recommended amount of daily fluid intake was 1.2 L/d for adults, but no recommendation was given for pregnant women [41]. In 2016, the recommendation was 2.7 L/d for women and 3.0 L/d for men and pregnant women [15]. However, the recommendation for water intake for pregnant women was based on data of the water intake survey among pregnant women in other countries, not the data in China. Water requirements vary among pregnant women in different countries due to differences in dietary pattern, environment, and other factors; thus, it is necessary to undertake studies related to water intake during pregnancy in China.

Two hypotheses are proposed in this study: one is that some pregnant women are dehydrated due to having insufficient water intake and the other is that dehydration has negative effects on pregnancy and maternal and infant outcomes. The objectives of this study are, firstly, to investigate the fluid intake among pregnant women, secondly, to assess their hydration state, and finally, to investigate the associations with pregnancy complications and maternal-infant outcomes. This preliminary exploratory study on will provides some related reference data for updating the recommendation for adequate water intake among pregnant women. In addition, it will provide some evidences on water-related education. Based on this preliminary study, multicenter studies with larger sample sizes are required for the development of clinical guidelines and recommendations. The ultimate goals are to promote adequate water intake, to maintain optimal hydration state, and to improve mater health and birth outcomes.

## Methods/design

### Study hypotheses

One hypothesis is that a certain proportion of pregnant women have insufficient water intake and have risk of being in dehydration state. Another hypothesis is that dehydration state induced by insufficient water intake affects maternal health and birth outcomes.

### Study design

A prospective observational cohort study is designed. A total of 380 pregnant women with < 13 weeks’ gestation will be recruited. All items from the World Health Organization Trial Registration Data Set are shown in Table 1.

**Table 1:** Items from the World Health Organization Trial Registration Data Set

Data Category	Information
Registration number	ChiCTR1800019284, Chinese clinical trial registry
Registration State	1,008,001 Prospective registration
Public title	Study for the correlation between hydration state and pregnancy complications, maternal and infant outcomes during pregnancy
Scientific title	The correlation between hydration state and pregnancy complications, maternal and infant outcomes during pregnancy
Approval of ethic committee	Ethical Review Committee of the Hainan Medical University
Ethical approval project identification code	2018–4
Date of approved by ethic committee	20 May 2018
Study type	Prospective observational study
Study design	Prospective observational study
Key inclusion and exclusion criteria	Inclusion criteria: Aged between 21~35; at first trimester of pregnancy (before 13 weeks), in health state, without metabolic disease, oral diseases, and so on. Exclusion criteria: Aged < 21, or age > 35; not being at first trimester of pregnancy (before 13 weeks); With metabolic disease, oral diseases, and other diseases.
Outcomes	Urine and blood osomolality, fluid intake, copeptin, pregnancy complications, maternal and infant outcomes
Collecting samples	Urine and blood
Recruitment state	Not initiated
Randomization Procedure (please state who generates the random number sequence and by what method)	Not applicable. A convenience sampling method will be used to recriute participants.

### Sample size calculation

For the calculation of sample size, the variable used is the incidence of new-onset hyperglycemia. Using the following formula, sample size is calculated:$$ {n}_1=\frac{\left(1+\raisebox{1ex}{$1$}\!\left/ \!\raisebox{-1ex}{$C$}\right.\right)\overline{p}\ \overline{q}{\left({U}_1-\raisebox{1ex}{$\alpha $}\!\left/ \!\raisebox{-1ex}{$2$}\right.+{U}_1-\beta \right)}^2}{{\left({P}_1-{P}_0\right)}^2} $$; *α* = 0.05, *β* = 0.20. Among these parameters, *p*_*1*_ = 20.9%, indicates the incidence of new-onset hyperglycemia among participants with insufficient fluid intake; *p*_*0*_ = 9.0%, means the incidence of new-onset hyperglycemia among participants with sufficient fluid intake; *c* = 4, indicates the ratio of pregnant women with water intake of > 0.5 L to pregnant women with water intake of < 0.5 L. These parameters were set based on the reference to the results of a related study [42]. A 10% dropout rate is taken into account to obtain the final sample size. For validity, 380 pregnant women will be needed.

### Participants

Pregnant women will be recruited from the First Affiliated Hospital of Hainan Medical University, Hainan province of China, using a convenience sampling method. The inclusion criteria are as follows: aged between 21 and 35 years; primigravidas; in the first trimester of pregnancy (before 13 weeks’ gestation); and without kidney diseases, diabetes mellitus, digestive system diseases, cardiovascular diseases and other diseases. The exclusion criteria are as follows: smoking; habitual consumption of alcohol (> 20 g/day) [43] or intensive physical activity; or with the diseases of kidney diseases, diabetes mellitus, digestive system diseases, cardiovascular diseases and other diseases.

### Ethics

The study protocol has been reviewed and approved by the Ethical Review Committee of the Hainan Medical University. Ethical approval project identification code is 2018–4. The study will be carried out according to the principles of the Declaration of Helsinki. All participants will read the informed consent form, voluntarily agree to participate in this study and sign the informed consent form prior to the study. Written informed consent will be obtained from each participants before enrolment in the study, and then preserved by researchers.

### Study procedure

**Fig. 1:**
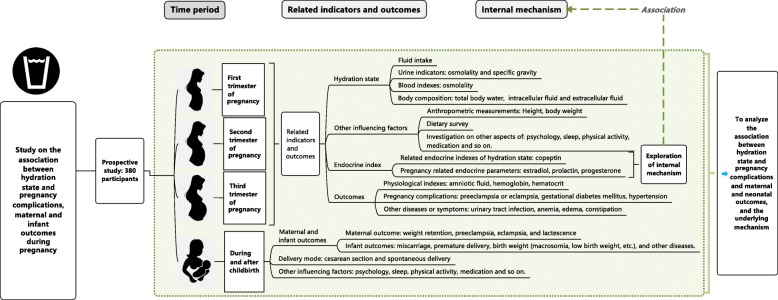
Technology roadmap of study

Participants will be followed throughout pregnancy to childbirth. And their infants will also be followed up to 42 days of age. Relevant indicators and outcomes will be measured and followed at the first trimester of pregnancy (15–17 weeks’ gestation), second trimester of pregnancy (20–22 weeks’ gestation), third trimester of pregnancy (30–32 weeks’ gestation), during childbirth and 42 days after childbirth (Fig. 1).

After recruitment: Maternal socio-economics, socio-demographics and other basic information, such as pregnancy history, childbearing history, family history, disease history, drug use and anthropometric data of the participants will be collected after being recruited and entry into the study.

During the first trimester of pregnancy: Survey of fluid intake, dietary intake, psychological state, sleep quality and time, physical activities, history of disease and medication-use will be conducted using corresponding questionnaires at 1 weeks before antenatal care. On the day of antenatal care, anthropometric measurements, such as height, body weight and body composition, will be assessed by obstetricians. The first morning urine samples will be collected in sterile disposable urine sample cup and urine osmolality will be tested immediately by professional laboratory technicians. Antecubital venous blood will be collected to test hemoglobin, osmolality, hydration state regulating endocrine hormones – copeptin and pregnancy related endocrine hormones, including estradiol, prolactin, and progesterone. Pregnancy complications and other diseases or symptoms including urinary tract infection, anemia, edema and constipation will be assessed and diagnosed by obstetricians in accordance with the relevant physiological indicators. The occurrence time, duration and treatment measures of these outcomes will be recorded in detail as an important factor to be considered in later data analysis.

During the second and third trimester of pregnancy: the same procedure as the first trimester of pregnancy.

**Fig. 2:**
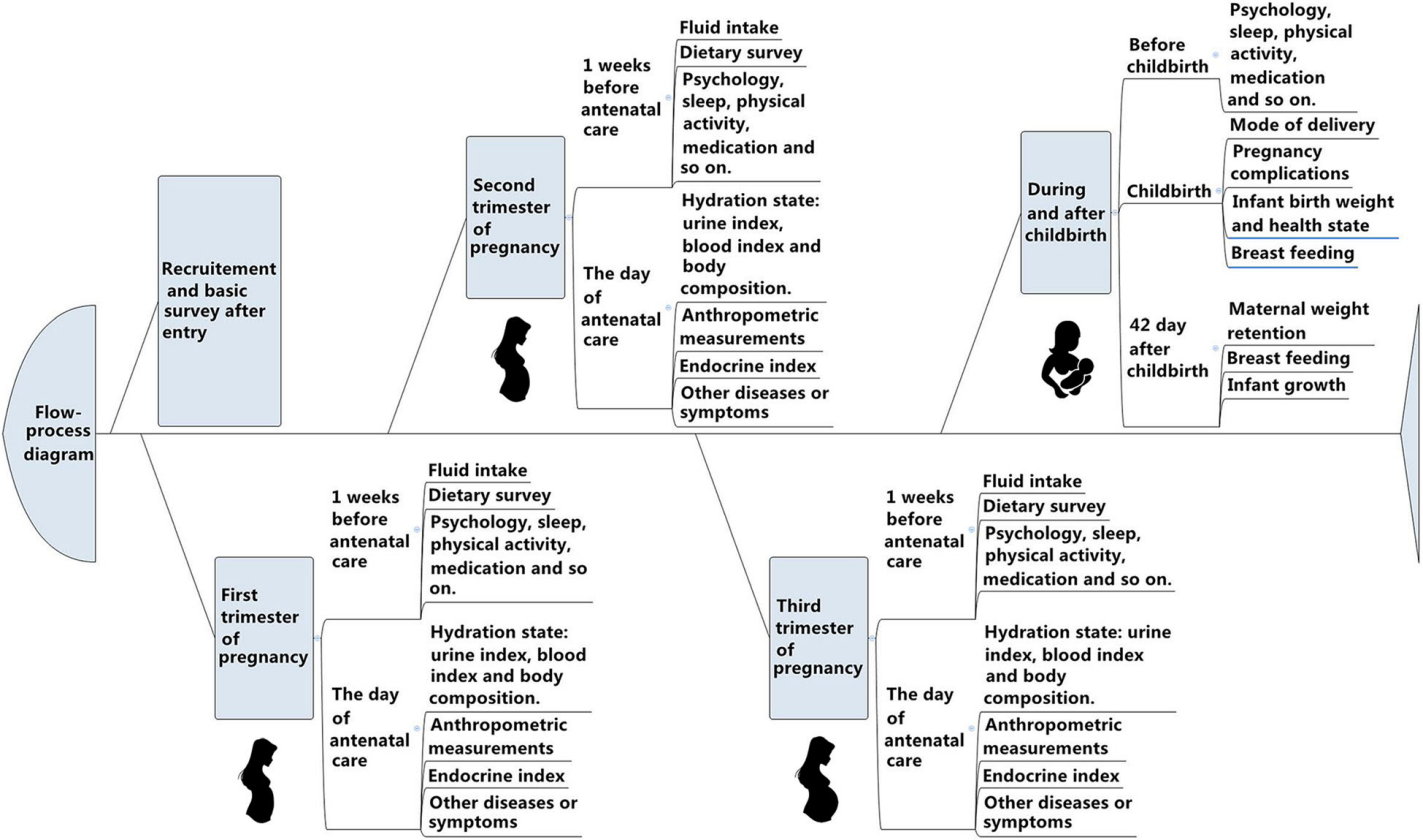
Flow-process diagram of the study

During childbirth and after childbirth: Before childbirth (One day before the expected date of delivery or one day before cesarean section), psychological state, sleep quality and time, physical activities and medication-use will be collected using corresponding questionnaires. On the day of childbirth, the mode of delivery will be recorded, pregnancy complications and other complications during childbirth will be assessed and diagnosed by professional obstetricians. Infant birth weight will be measured and health state will be assessed. Breastfeeding time and the crying behavior of infants before and after breast-feeding will be recorded. At 42 days after childbirth, maternal weight retention will be measured and calculated. Secretion of breast milk and mode of infant feeding will be consulted and infant growth will be assessed. Flow-process diagram of the study was shown in Fig. 2.

If participants in this study requires hospitalization for any reason or receives intravenous fluid, or develops vomiting or diarrhea or fever or respiratory tract infections during pregnancy, the occurrence time, duration and treatment measures of these incidents will be recorded in detail. The time for recording fluid intake, food intake and other indicators will be exchanged within the allowable range of corresponding research phase to stagger the occurrence and duration of these situations.

The schedule of enrolment, data collections and assessments are shown in Table 2.

**Table 2:** The schedule of enrolment, interventions, and assessments

Timepoint	15 April 2019	15 April 2019–30 September 2020	1 Octorber 2020
Enrolment:			
Eligibility screen	√	√	
Informed consent	√	√	
Collection of basic information	√	√	
Assessments of related indicators and outcomes during pregnancy and childbirth	√	√	
Close-out			√

### Hydration related indicators and maternal-infant outcomes

Hydration related indicators and maternal-infant outcomes are summarized in Table 3.

**Table 3:** Summarize of hydration related indicators and maternal-infant outcomes in this study

Related indicators and outcomes	Trimester of pregnancy			
	First trimester (15–17 weeks)	Second trimester (20–22 weeks)	Third trimester (30–32 weeks)	During and after childbirth
Related indicators				
Fluid intake	√	√	√	√
Dietary survey	√	√	√	√
Other influencing factors	√	√	√	√
Endocrine index	√	√	√	
Hydration state	√	√	√	√
Outcomes				
Physiological index	√	√	√	
Pregnancy complications	√	√	√	√
Other diseases or symptoms	√	√	√	√
Delivery mode				√
Maternal weight intention				√
Breast feeding				√
Infant weight				√

### Definition of hydration

Hydration state is determined by the balance between water inputs and outputs and judged by the standard of urine osmolality. Dehydration is judged as urine osmolality of > 800 mOsm/kg, and it occurs when water inputs is insufficient to replace water outputs [44]. Middle hydration state is judged as urine osmolality between 500 and 800 mOsm/kg [45]. Optimal hydration state is judged as urine osmolality of ≤500 mOsm/kg [45].

### Assessment of indicators and outcomes

#### Fluid intake

After standardized training by researchers, daily fluid intake of participants will be collected using a 7-day 24-h fluid intake record by themselves for correct use of such records. The amount of fluid intake for each time in seven consecutive days will be measured using a customized cup, and the nearest of cup scale is10 mL. This method has been proved to be reliable in many water intake-related studies [21, 46, 47]. The volume, type, time and place of water intake will also be recorded by participants. All types of fluid intake will be recorded in detail including plain water, bottled water, tea, sugar-sweetened beverages, and so on. The record will be photographed and sent by participants to the investigators using a mobile phone, and will be reviewed by investigators every day to ensure the accuracy and the integrity of the records.

#### Food intake and water intake from food

Food intake and water intake from food will be recorded and estimated using a semi-quantitative food frequency questionnaire (FFQ). The type, frequency and amount of food intake will be recorded. The amount of food intake will be estimated using by participants with the references of food models and photographic models [48]. Nutrients and water intake from food will be assessed by trained investigators using the *Chinese Food Composition Table* [49].

#### Psychological status:-anxiety and depression

Self-rated anxiety scale and self-rating depression scale will be used as indicators to assess the psychological status of participants [50–52]. Both scales, which mainly evaluates the frequency of symptoms, have 20 items each. Frequency will be rated on a 4-point scale: 1 indicates “never or seldom,” 2 indicates “sometimes,” 3 indicates “most of the time,” and 4 indicates “all the time”. For negative items, scores are graded sequentially according to the order from 1 to 4, and vice versa. The T score = total score for 20 items× 1.25 (the decimal portion is rounded). Standard of assessing anxiety: A T score 50–59 means mild anxiety, 60–69 means moderate anxiety, > 69 means severe anxiety, and < 50 means normal. Standard of assessing depression: T score 53–62 means mild anxiety, 63–72 means moderate anxiety, > 72 means severe anxiety, and < 53 means normal.

#### Sleep quality and time

The Pittsburgh Sleep Quality Index (PSQI) will be used to assess sleep quality and time for participants, which include 19 self-rating items and 5 others-rating items [53, 54]. Score is calculated including 19 self-rating items. Total 7 factors are measured in the 19 items: subjective sleep quality; sleep latency; sleep persistence; habitual sleep efficiency; sleep disorder; use of sleep drugs and daytime dysfunction. A 4-point scale is used, “0” means “no difficulty”; “2” means “moderate difficulty”; “3” means “severe difficulty”; and “1” means “mild difficulty”. The higher the score, the poorer the quality of sleep.

#### Physical activity

Information on physical activity will be collected by using the International Physical Activity Questionnaire (IPAQ) [55, 56]. The IPAQ consists of 27 items, incluing the type, frequency and duration of various physical activities. Participants will be classified into low-intensity, moderate-intensity and vigorous-intensity physical activity according to the similar criteria in some references [57, 58].

#### Anthropometric measurements

On the day of antenatal care, height and weight will be measured twice by professional obstetricians using the height-weight meter (HDM-300; Huaju, Yiwu, Zhejiang, China) following standardized processes to the nearest 0.1 cm and 0.1 kg, respectively. [BMI: weight (kg)/height squared (m)].

#### Indicators related to hydration state

About the evaluation criteria and indicators for hydration state, multiple indicators will be used for comprehensive evaluation, so body fluids, fluid intake, urine and blood osmolality are measured for evaluating hydration state in this study. At present, the internationally recognized and authoritative indicator is urine osmolality, which will be used as the primary determinant of the hydration state in this analysis of this study, and other indicators will be considered to be auxiliary indicators.

Intracellular fluid (ICF) and extracellular fluid (ECF): It will be measured by professional obstetricians using a body composition analyzer (Inbody 720; Inbody; Seoul, Korea) with the patients in a fasting state and after having defecated and urinated [59–61].

Urine and blood osmolality: The first morning urine samples will be collected in a sterile disposable urine sample cup, and blood will also be collected in vacuum tubes. Using an osmotic pressure molar concentration meter (SMC 30C; Tianhe, Tianjin, China) with the freezing-point method, osmolality will be determined by laboratory physicians, which will be used to assess the hydration state of participants.

Urine-specific gravity (USG): Using an automatic urinary sediment analyzer (FUS-200, Dirui, Changchun, China) with the uric dry-chemistry method, the first morning urine samples will be collected in sterile disposable urine sample cup to determine USG by laboratory physicians.

#### Endocrine indexes related to hydration and pregnancy

Copeptin as a hydration regulatory endocrine index and estradiol, prolactin, progesterone as pregnancy-related endocrine indexes will be tested in this study. The elbow venous blood will be collected in the morning and injected into a centrifugal tube containing 0.3 mol/L EDTA-Na2 and 5 × 10^5^ units of aprotinin. The supernatant was centrifuged at 3000 rpm/min for 15 min and stored at − 20 C. Then, these endocrine indexes will be determined with the method of radioimmunoassay with corresponding kits.

#### Pregnancy complications, other diseases or symptoms and maternal outcomes

Gestational hypertension: Blood pressure and will be measured twice by obstetricians to the nearest 2 mmHg with the desktop mercury sphygmomanometer (Yuwell, Danyang, Jiangsu, China). Two measurements will be taken after 2-min intervals. Hypertension of pregnancy will be defined as systolic blood pressure of > 140/90 mmHg and/or diastolic blood pressure of ≥90 mmHg sustained on two measurements.

Gestational diabetes mellitus (GDM): Blood glucose will be determined with elbow venous blood using an osmotic pressure molar concentration meter (SMC 30C; Tianhe, Tianjin, China) by laboratory physicians. GDM will be diagnosed based on the results of the 100-g, 3-h oral glucose tolerance test (OGTT) by obstetricians. According to the recommendations of the Committee on Obstetric Practice, a definite diagnosis is made if two or more thresholds be met or exceeded. The threshold of fasting blood glucose is as follows:5.3 mmol/L, 10.0 mmol/L for blood glucose of 1 h, 8.6 mmol/L for blood glucose of 2 h and 7.8 for blood glucose of 3 h [62].

Preeclampsia or eclampsia: Urine protein will be assessed using an automatic biochemical analyzer (Cobas C501; Roche; Basel, Switzerland) by laboratory physicians. Preeclampsia will be diagnosed at ≥20 weeks’ gestation along with new-onset gestational hypertension and new-onset proteinuria [63]. Eclampsia will be defined as the development of convulsions and/or unexplained coma during or after pregnancy with symptoms of preeclampsia, which will be diagnosed by obstetricians [64].

Anemia: Hemoglobin of elbow venous blood will be tested using automatic routine blood test device (MC-600, Kubeier, Shenzhen, China) by laboratory physicians. Anemia will be defined as < hemoglobin 110 g/L.

Oligohydramnios: Amniotic fluid will be measured using ultrasonography by professional medical technicians. Oligohydramnios is diagnosed by an amniotic fluid index (AFI) of < 5 cm or maximum amniotic fluid pool depth of < 2 cm [65].

Urinary tract infection: The diagnostic criteria is based on symptoms or laboratory confirmation including white blood cells and bacteria in urine by obstetricians. Routine urine test will be conducted using an automatic biochemical analyzer (Cobas C501; Roche; Basel, Switzerland) by laboratory obstetrician.

Spontaneous abortion: It will be recorded by obstetricians.

Preterm labor: It is defined as giving birth between 28 weeks’ and less than 37 weeks’ gestation. It will be diagnosed by obstetricians.

Mode of delivery: There are two modes: namely natural childbirth and cesarean section, which will be recorded by obstetricians.

Breast milk: The time for early initiation of breastfeeding will be recorded [66]. And the amount of breast milk will be evaluated based on the performances of infant after breast feeding.

Functional constipation: According to the Rome III criteria, it will be defined when participants present at least two of the following symptoms: at least one quarter of defecations for the last 12 weeks: lumpy or hard stools; straining; sensation of incomplete evacuation; sensation of anorectal obstruction; manual maneuvers to facilitate defecation; fewer than three defecations per week. Loose stools are rarely present without the use of laxatives [67]. Functional constipation will be diagnosed by obstetricians.

Total gestational weight gain (GWG) and postpartum weight retention (PWR): Both of them will be calculated based on weight of participants. GWG (kg) = weight before pregnancy (kg) -weight before delivery (kg). GWG will be assessed according to the recommendation of Institute of Medicine (IOM) [68]. PWR (kg) = weight before pregnancy (kg) - weight at 42 days after childbirth (kg).

#### Infant outcomes

Infant birth weight and length: They will be measured using length and weight-measuring devices for infants (HLZ-20; Hualizheng, Tianjin, China) by professional obstetricians while infant is wearing light clothing. The scales for weight and length are calibrated at intervals of 10 g and 0.5 cm, respectively. Low birth weight for newborn infants is defined as a weight of < 2.5 kg, while macrosomia is defined as a weight of ≥4 kg. The growth of infant will be assessed according to the 2006 WHO Child Growth Standards [69].

### Temperature and humidity of the environment

The temperature and humidity will be recorded according to the report of China Meteorological Administration at 9:00 AM and 3:00 PM each day.

### Confidentiality and withdrawal

The information confidential of the participants will be kept carefully in the whole process of study. During and after the trial, the names of participants will be replaced as study ID. Participants can withdraw from the trial freely as they wish. In case of adverse events, an emergency physician will be assigned to deal with them in time.

### Data entry and statistical analysis

*Data entry*: All data will be documented twice by two trained researchers using the software of Epi Data 3.1 to ensure the accuracy of data.

*Statistical analysis*: SAS 9.2 (SAS Institute Inc., Cary, NC, USA) will be used. A mixed model of repeated-measures (ANOVA) will be used to analyze the differences of outcomes among participants with different hydration state. Multi-logistic regression analysis will be used to investigate the influencing factors related to maternal health and infant outcomes, such as fluid intake, hydration state, urine osmolality, concentration of copeptin and other indicators. The level of significance will be set at 0.05 (*p* < 0.05, 2-tailed) with 95% confidence intervals (95% CI).

## Discussion

Pregnant women may need more fluid intake due to physiological changes in the mother and for fetal growth. Thus, the risk of being dehydration for pregnant women, due to insufficient fluid intake, is relatively high. However, surveys on fluid intake among pregnant women are scarce. Optimal hydration is essential for maintaining health. For pregnant women, the endocrine system shows considerable changes during pregnancy, which may affect water metabolism and balance and thus, hydration state. However, the importance of water and hydration, especially among pregnant women, is often neglected, and more research is required. In the present prospective cohort study, we will evaluate the fluid intake and hydration status of pregnant women and investigate the associations of fluid intake and hydration with pregnancy complications and maternal/infant outcomes.

The most considerable challenges in this study is the following up of participants and quality control. Important protocol modifications will be discussed and determined by the researcher group, and will be communicated to investigators, trial participants and other relevant parties by e-mail immediately. In the study, participants will be followed up from the first trimester of pregnancy to 42 days after childbirth. To increase the rate of follow up, effective and convenient methods of communication between participants and researchers will be established, including telephone call, home visits, e-mail, WeChat, short message and so on. The content and purpose of the study will be clearly clarified to establish a relationship of trust and raise awareness of participants concerning the importance of the study. In this study, many methods will also be used to ensure the quality control. The requirements for participants will be fully explained. Participants will also be trained to be familiarized with the content of related questionnaires and study requirements. If a participant failed to follow the requirements, her data will not be included in this study and the reason for dropout will also be recorded. Researchers will be trained on the procedure of this trial, the content of the questionnaires and the technology-related laboratory tests. Some indicators such as height, weight, and blood pressure will be measured twice for accuracy, such as height, weight and blood pressure. Food models and photographic models will be provided to help participants fill the questionnaire related to food and fluid intake after training to enhance estimation accuracy. Physiological indexes, such as blood and urine indicators, will be tested immediately after collection, in order to ensure the accuracy, and will be tested by laboratory physicians with the minimum 5 years of clinical experience to ensure accuracy. Trial conduction will be audited by a competent authority in the hospital every day, and the process will be independent from investigators and the sponsor. All the questionnaires will be regularly checked and stored for researcher rechecks. A data monitoring committee (DMC) is established and is comprised by six members (three clinicians, one statistician, one ethicist and one nutritionists), who are independent of the sponsor and competing interests. DMC will review the procedure and data of the study for once a week. The six members will evaluate the safety concerns and benefits to decide that if the study should be continued or terminated. They will also examine the quality of the data, supervise the quality control, record the follow-up process and supervise the process of data entry and analysis. Four hydration and fluid intake related projects has been completed in China by our team; these provided an authoritative questionnaire and technical supports for the study.

There are both strengths and weaknesses for the design of the present study. Referring to strengths, first, to eliminate the potential confounding factors as much as possible, age, BMI, physiological state, sleep quality, food intake and physical activity will be evaluated and included as covariates in the statistical analyses. Second, fluid intake is dependent on environmental factors, such as temperature and humidity. The conduction of this study will be in Hainan, which is an area with a tropical monsoon climate. Thus, temperature and humidity will be recorded clearly to actualize the comparability among different studies in China and with those in other countries. Third, hydration state will be evaluated in many indicators, not only including urine osmolality, but also including body compositions (ICF and ECF), copeptin, fluid intake and blood osmolality. Fourth, water intake from food will be assessed in order to calculate total water intake. Fifthly, the prospective cohort design can avoid recall bias and improve the accuracy of the results. In terms of weakness, the mechanism about the associations of hydration with pregnancy complications, maternal and infant outcomes will not be studied comprehensively. In this study, only the endocrine mechanisms related to hydration and pregnancy will be explored. An observational study will elucidate the associations but is not necessary for the development of recommendations unless confounding factors are carefully controlled.

In summary, it is the first time to assess water intake and hydration stare among pregnant women, and it is the first time to investigate the associations of hydration with pregnancy complications, maternal and infant outcomes in China. Our results can provide some scientific reference data for updating the recommendations on fluid take for pregnant women and valuable evidence on the importance of hydration on maternal health during pregnancy. The results may also raise awareness on the importance of adequate fluid intake and optimal hydration in Chinese residents, especially pregnant women. More multicenter studies with larger sample sizes are required for the development of clinical guidelines and recommendations. The final goal is to develop water intake–related interventions to improve maternal health and birth outcomes.

## Data Availability

No additional data are available for protocol. The datasets used and/or analyzed of the study will be available from the corresponding author on reasonable request after finishing the study.

## Abbreviations

ECF: Extracellular fluid
FFQ: Food frequency questionnaire
GDM: Gestational diabetes mellitus
GWG: Total gestational weight gain
ICF: Intracellular fluid
IPAQ: International Physical Activity Questionnaire
OGTT: Oral glucose tolerance test
PSQI: Pittsburgh Sleep Quality Index
PWR: Postpartum weight retention
USG: Urine-specific gravity
